# The Effect of Dexmedetomidine on Emergence Agitation or Delirium in Children After Anesthesia—A Systematic Review and Meta-Analysis of Clinical Studies

**DOI:** 10.3389/fped.2020.00329

**Published:** 2020-07-14

**Authors:** Yuquan Rao, Ruifeng Zeng, Xuebin Jiang, Jun Li, Xiaocou Wang

**Affiliations:** Department of Anesthesiology, Critical Care and Pain Medicine, The Second Affiliated Hospital, Wenzhou Medical University, Wenzhou, China

**Keywords:** dexmedetomidine, pediatric, agitation, delirium, meta-analysis

## Abstract

**Background:** We conducted this systematic review and meta-analysis to investigate the clinical effect of dexmedetomidine in preventing pediatric emergence agitation (EA) or delirium (ED) following anesthesia compared with placebo or other sedatives.

**Methods:** The databases of Pubmed, Embase, and Cochrane Library were searched until 8th January 2020. Inclusion criteria were participants with age<18 years and studies of comparison between dexmedetomidine and placebo or other sedatives. Exclusion criteria included adult studies; duplicate publications; management with dexmedetomidine alone; review or meta-analysis; basic research; article published as abstract, letter, case report, editorial, note, method, or protocol; and article presented in non-English language.

**Results:** Fifty-eight randomized controlled trials (RCTs) and five case-control trials (CCTs) including 7,714 patients were included. The results showed that dexmedetomidine significantly decreased the incidence of post-anesthesia EA or ED compared with placebo [OR = 0.22, 95% CI: (0.16, 0.32), *I*^2^ = 75, *P* < 0.00001], midazolam [OR = 0.36, 95% CI: (0.21, 0.63), *I*^2^ = 57, *P* = 0.0003], and opioids [OR = 0.55, 95% CI: (0.33, 0.91), *I*^2^ = 0, *P* = 0.02], whereas the significant difference was not exhibited compared with propofol (or pentobarbital) [OR = 0.56, 95% CI: (0.15, 2.14), *I*^2^ = 58, *P* = 0.39], ketamine [OR = 0.43, 95% CI: (0.19, 1.00), *I*^2^ = 0, *P* = 0.05], clonidine [OR = 0.54, 95% CI: (0.20, 1.45), *P* = 0.22], chloral hydrate [OR = 0.98, 95% CI: (0.26, 3.78), *P* = 0.98], melatonin [OR = 1.0, 95% CI: (0.13, 7.72), *P* = 1.00], and ketofol [OR = 0.55, 95% CI: (0.16, 1.93), *P* = 0.35].

**Conclusion:** Compared with placebo, midazolam, and opioids, dexmedetomidine significantly decreased the incidence of post-anesthesia EA or ED in pediatric patients. However, dexmedetomidine did not exhibit this superiority compared with propofol and ketamine. With regard to clonidine, chloral hydrate, melatonin, and ketofol, the results needed to be further tested due to the fact that only one trial was included for each control drug.

## Introduction

Emergence agitation (EA) or delirium (ED) manifests as a series of sudden complex psychomotor disorders, characterized by perceptual disturbances, delusions, and disorientation following sedation or general anesthesia (1). So far, the specific mechanism of EA or ED has not been clear. The preschool children undergoing ophthalmology or otorhinolaryngology procedures under inhalation agents are susceptible population (2). According to some studies, the incidence of EA or ED after general anesthesia in children ranges from 10 to 80% (3) and significantly increases the occurrence of other complications after anesthesia, like self-injury, prolonged post-anesthesia care unit (PACU) stay, poor satisfaction of parents and care providers, and so on (4). Therefore, it is necessary to find effective measures to prevent or treat EA or ED.

Some studies have reported the pharmacological strategies to prevent EA or ED, including midazolam, propofol, ketamine, opioids, and α_2_ adrenergic receptor agonists (5–8). Activation of an α_2_ adrenergic receptor can contribute to pharmacological effects of sedation, analgesia, and anti-inflammation; thus, an α_2_ adrenergic receptor may be a target for prevention and treatment of EA or ED (9, 10). A study from Ydemann et al. (11) found that clonidine significantly decreased the incidence of postoperative agitation in children after sevoflurane anesthesia compared with placebo. Another commonly used α_2_ adrenergic receptor agonist dexmedetomidine shows a higher ratio of specificity for α_2_ receptor (α_2_:α_1_ 1600:1) compared with clonidine (α_2_:α_1_ 200:1) (12, 13). Although dexmedetomidine is used as an off-label drug in children, increasing studies about the effect of dexmedetomidine on EA and ED in pediatric patients have been completed. We conducted this meta-analysis for clinical trials to evaluate the effect of dexmedetomidine on EA or ED following sedation or general anesthesia in pediatric patients compared with placebo and other drugs.

## Materials and Methods

This systematic review and meta-analysis was performed according to the guidelines of the 2009 PRISMA (Preferred Reporting Items for Systematic reviews and Meta-analyses) (Supplementary Table 1) (14).

### Search Strategy and Study Selection

We searched the databases including “Pubmed,” “Embase,” and “Cochrane Library” through the PICOS (Population, Intervention, Comparison, Outcome, Study design) method until 8th January 2020. The entry words included “child” OR “children” OR “pediatric” AND “dexmedetomidine” OR “precedex” OR “MPV-1440” OR “MPV 1440” OR “Dexmedetomidine Hydrochloride” OR “Hydrochloride, Dexmedetomidine” AND “agitation” OR “delirium,” and the search scope was “all fields.” Because all studies about the effect of dexmedetomidine vs. other drugs (placebo or other sedatives) on agitation or delirium in pediatric patients were eligible in this meta-analysis, we did not confine the search words of control drugs and study design. The inclusion criteria included the following: (1) participants with age<18 years; and (2) management with prophylactic dexmedetomidine and placebo or other sedatives. The exclusion criteria included the following: (1) participants with age≥18 years; (2) management with dexmedetomidine alone; (3) review or meta-analysis; (4) basic research; (5) article published as an abstract, letter, case report, editorial, note, method, or protocol; and (6) article presented in non-English language.

### Data Analysis

The aim of this meta-analysis was to investigate whether dexmedetomidine had advantage in reducing the incidence of EA or ED following sedation or general anesthesia in pediatric patients compared with placebo or other sedatives.

Three authors were independently responsible for reviewing the titles, abstracts, or both and summarized the data of the included literatures. Another two authors were in charge of the data discrepancy adjustment.

Two authors were responsible for extracting the following information: (1) authors; (2) publication year; (3) number of the total participants in each study; (4) age range of all the participants; (5) country of publication; (6) procedures that the participants underwent; (7) time of dexmedetomidine or other sedative administration; (8) infusion speed or dosage of dexmedetomidine or other sedatives; and (9) number of patients with EA or ED following sedation or general anesthesia.

Two authors independently assessed the quality of included studies. The risk of bias of randomized controlled trials (RCTs) were assessed by the Cochrane Collaboration Risk of Bias Assessment tool including seven items: random sequence generation, allocation concealment, blinding of participants and personnel, blinding of outcome assessment, incomplete outcome data, selective reporting, and others (bias due to vested financial interest and academic bias). If a trial had one or more of the items to be judged as high or unclear risk of bias, this trial was classified as having high risk (15). The bias risk of case-control trials (CCTs) was assessed by the Newcastle-Otawa Quality Assessment Scale (NOS) comprising three domains: selection, comparability, and outcome for cohort studies. There were four stars in the selection domain, two stars in the comparability domain, and three stars in the exposure domain. Trials with cumulative seven stars or more were considered to be of high quality, those with six stars were considered to be of moderate quality, and those with less than six stars were considered to be of low quality (Supplementary Table 2) (16). If the two authors had different assessment results, they consulted the third or the fourth one. Eventually, the authors reached consensus. All included trials were grouped based on different control drugs.

RevMan Review Manager version 5.3 (Cochrane collaboration, Oxford, UK) and Stata version 12.0 (Stata Corp, College Station, TX, USA) were used to perform statistical analyses. The values of *I*^2^ and the Mantel–Haenszel chi-square test (*P*-value for heterogeneity) were used to evaluate the heterogeneity of included studies. And the values of *I*^2^ < 40%, 40–60%, and >60% represented low, moderate, and high heterogeneity, respectively (17). If *I*^2^ > 50% or a *P*-value for heterogeneity <0.1 was identified, the method of random-effect model analysis was applied to analyze the data. Conversely, if *I*^2^ < 50% or a *P*-value for heterogeneity ≥0.1 was presented, the method of a fixed-effect model was used (18). The dichotomous outcome was reported as odds ratios (OR) with 95% confidence interval (CI). The statistical tests were two-sided, and a *P*-value for overall effect <0.05 was considered to have significant difference.

Sensitivity analysis was conducted to solve the problem of significant heterogeneity (*I*^2^ > 40%) through the method of subgroup analysis or one-by-one literature removal. Meta-regression was used to investigate the heterogeneity sources for the group with *I*^2^ > 40% according to possible risk factors. A subgroup analysis proceeded based on the risk factor with *P* < 0.05 by meta-regression analysis; conversely, the method of one-by-one literature removal was used if *P*-values of all risk factors were 0.05 or more.

## Results

### Study Location and Selection

**Figure 1:**
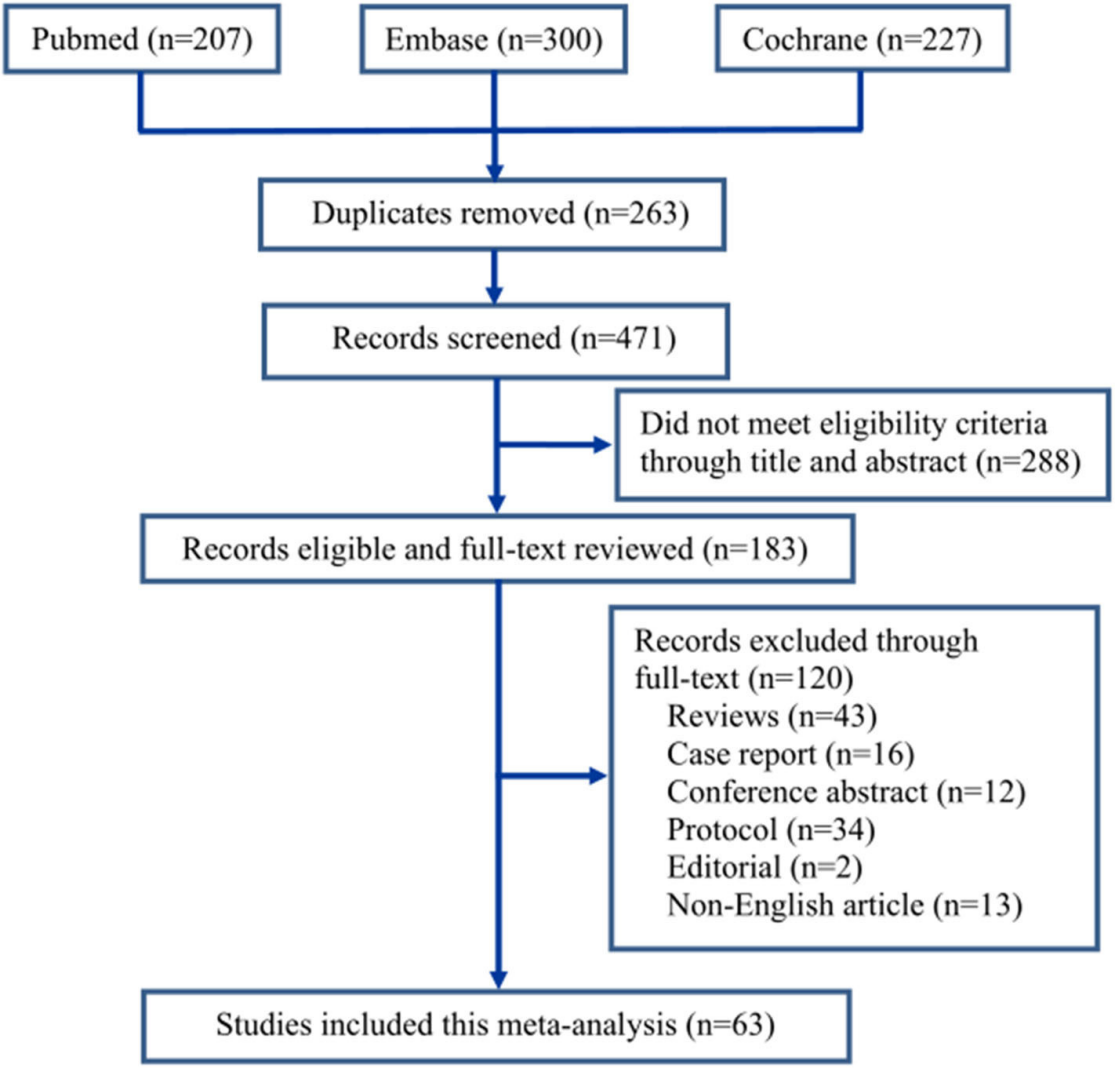
The flow chart of study screening.

The screening process of the eligible literatures is shown in Figure 1. We obtained 207 trials from Pubmed, 300 from Embase, and 227 from Cochrane Library according to the inclusion criteria. Two hundred sixty-three trials were removed due to duplicates. Two hundred eighty-eight trials were excluded because they did not meet the eligibility criteria by browsing the titles and abstracts, and 120 trials were removed by browsing the full text. Eventually, 63 trials (19–81) including 7,714 patients were identified through our search strategy (Figure 1). All included trials were divided into nine groups based on control drugs: placebo (19–59), midazolam (19, 59–71), opioids (29, 45, 72–74), propofol (or pentobarbital) (22, 25, 42, 75, 76), ketamine (26, 60, 77–79), clonidine (80), chloral hydrate (81), melatonin (59), and ketofol (ketamine and propofol) (23). We assigned propofol and pentobarbital into the same group because both of them produced general anesthetic efficacy through directly activating the γ-aminobutyric acid A receptor of the central nervous system (82).

### Characteristics of Included Trials

There were 41 trials (19–59) including 3,600 patients in the placebo group, 14 trials (19, 59–71) including 1,033 patients in the midazolam group, 5 trials (29, 45, 72–74) including 396 patients in the opioids group, 5 trials (22, 25, 42, 75, 76) including 1,969 patients in the propofol (or pentobarbital) group, 5 trials (26, 60, 77–79) including 332 patients in the ketamine group, and 4 trials (23, 59, 80, 81) including 384 patients in the clonidine, chloral hydrate, melatonin, and ketofol group, respectively.

Table 1 demonstrated the basic information of all included trials; meanwhile, it was discovered that clinical heterogeneity might be associated with the study methods, the type of surgery, the number and age of participants, and the route, dosage, and timing of drug administration. Five trials in the included studies were CCTs (36, 46, 65, 66, 76). The patients in 17 trials (20, 22, 28–30, 32, 39, 43, 49, 50, 52, 54, 61, 64, 72, 74, 78) underwent otolaryngology surgeries, those in 5 trials (36, 44, 66, 67, 70) underwent dental or cleft palate surgeries, those in 9 trials (19, 26, 38, 40, 53, 55–57, 63) underwent ophthalmic surgeries, those in 11 trials (21, 27, 33, 34, 37, 41, 42, 47, 68, 69, 71) underwent general or urological surgeries, those in 4 trials (23, 31, 62, 73) underwent orthopedic surgeries, those in 1 trial (65) underwent cardiac surgery, those in 8 trials (24, 45, 46, 58–60, 75, 79) underwent invasive examination or treatment, those in 5 trials (25, 35, 76, 77, 81) underwent non-invasive examination or treatment, and those in 3 trials (48, 51, 80) underwent all kinds of outpatient surgeries. Different routes of drug administration were used: intranasal in 12 trials (19, 24, 28, 40, 45, 56, 58, 61, 71, 78, 80, 81), oral in 5 trials (36, 66–69), caudal or nerve block in 5 trials (21, 41, 47, 57, 73), inhalation in 1 trial (60), transmucosal in 1 trial (20), and intravenous in 39 trials. The strategy of drug administration was also different: (1) intravenous single dose in 20 trials (22, 23, 25, 29, 30, 32–35, 42, 48–50, 53–55, 59, 63, 64, 72), loading dose plus maintenance infusion in 10 trials (26, 31, 37, 43, 52, 74–77, 79), and only maintenance infusion in 6 trials (38, 39, 44, 51, 62, 65); and (2) administration onset before anesthesia in 29 trials (19, 20, 24, 28, 36, 40, 45, 46, 49, 55, 56, 58–61, 63, 66–71, 75–81), during anesthesia in 32 trials (21–23, 25–27, 29–35, 37–39, 41–44, 47, 48, 50–54, 57, 64, 72–74), and after anesthesia in 2 trials (62, 65). The number of patients with EA or ED in dexmedetomidine and control groups is shown in Table 2.

**Table 1:** The basic information of all included trials.

**Study**	***N***	**Age**	**Center/ country**	**Procedures**	**Anesthesia**	**Dexmedetomidine dosage**	**Control**
Ibacache et al. (34) (Prospective)	90	1–10 years	Single/Chile	Inguinal hernia repair, orchiopexy, or circumcision	General anesthesia (sevoflurane)+ caudal block	After induction, 0.15 μg/kg intravenously in 10 min After induction, 0.3 μg/kg intravenously in 10 min	Placebo (normal saline)
Guler et al. (30) (Prospective)	60	3–7 years	Single/Turkey	Adenotonsillectomy	General anesthesia (sevoflurane)	About 5 min before the end of surgery, 0.5 μg/kg was infused over a time period of 5 min	Placebo (normal saline)
Shukry et al. (51) (Prospective)	46	1–10 years	Single/USA	Outpatient surgical procedures	General anesthesia (sevoflurane)	5 min following securing the airway, dexmedetomidine was infused at a dose of 0.2 μg/kg/h	Placebo (normal saline)
Isik et al. (35) (Prospective)	42	18 months to 10 years	Single/Turkey	Cranial MRI scanning	General anesthesia (sevoflurane)	1 μg/kg was infused over 2 min after the induction	Placebo (normal saline)
Erdil et al. (29) (Prospective)	90	2–7 years	Single/Turkey	Adenoidectomy	General anesthesia (sevoflurane)	Dexmedetomidine 0.5 μg/kg after tracheal intubation	Fentanyl: 2.5 μg/kg after tracheal intubation Placebo (normal saline)
Saadawy et al. (47) (Prospective)	60	1–6 years	Single/Egypt	Unilateral inguinal hernia/orchidopexy	General anesthesia (sevoflurane)+caudal block	Dexmedetomidine 1 μg/kg with bupivacaine caudal block after induction	Placebo with bupivacaine caudal block
Talon et al. (71) (Prospective)	100	1–18 years	Single/USA	Elective reconstructive surgery for cutaneous burn injuries	General anesthesia (sevoflurane)	Tranasal dexmedetomidine 2 μg/kg	Oral midazolam 0.5 mg/kg
Koruk et al. (79) (Prospective)	18	2–13 years	Single/Turkey	Transcatheter atrial septal closure operation	General anesthesia (propofol)	Loading: 1 μg/kg was given over 10 min, followed by a dose of 0.5 μg/kg/h before anesthesia	Loading: Ketamine 1 mg/kg 10 min, followed by a rate of 0.5 mg/kg/h
Patel et al. (74) (Prospective)	122	2–10 years	Single/USA	Adenotonsillectomy	General anesthesia (sevoflurane)	Loading: 2 μg/kg over 10 min, followed by 0.7 μg/kg/h until 5 min before the end of the surgery	Intravenous fentanyl (1 μg/kg) as a bolus as soon as intravenous access was obtained
Sato et al. (48) (Prospective)	81	1–9 years	Single/Japan	Outpatient surgical procedures	General anesthesia (sevoflurane)	0.3 μg /kg dexmedetomidine was infused over 10 min after induction of anesthesia	Placebo (normal saline)
Bedirli et al. (72) (Prospective)	77	2–12 years	Single/Turkey	Adenotonsillectomy	General anesthesia (sevoflurane)	1 μg/kg dexmedetomidine after intubation	2 mg/kg tramadol after intubation
Mason et al. (76) (Retrospective)	1662	0.5–5.7 years	Single/USA	CT scanning	Sedation	Loading 2 μg/kg administered over 10 min, followed by infusion of 1 μg/kg/h is initiated and maintained until completion of the imaging study	Pentobarbital 2–3 mg/kg
Mountain et al. (67) (Prospective)	41	1–6 years	Single/USA	Dental restoration and possible tooth extraction	Sedation	4 μg/kg of oral dexmedetomidine	0.5 mg/kg of oral midazolam
Özcengiz et al. (59) (Prospective)	100	3–9 years	Single/Turkey	Esophageal dilatation procedures	General anesthesia (sevoflurane)	Dexmedetomidine 2.5 μg/kg before induction of anesthesia	Midazolam 0.5 mg/kg Melatonin 0.1 mg/kg Placebo (normal saline)
Pestieau et al. (45) (Prospective)	101	6 months to 6 years	Single/USA	Insertion of pressure-equalizing tubes	General anesthesia (sevoflurane)	Intranasal dexmedetomidine 1 μg/kg Intranasal dexmedetomidine 2 μg/kg	Intranasal fentanyl 2 μg/kg Acetaminophen
Akin et al. (61) (Prospective)	90	2–9 years	Single/Turkey	Adenotonsillectomy	General anesthesia (sevoflurane)	1 μg /kg was used intranasally 45–60 min before the induction of anesthesia	0.2 mg/kg midazolam was used intranasally 45–60 min before the induction of anesthesia
Meng et al. (43) (Prospective)	120	5–14 years	Single/China	Tonsillectomy	General anesthesia (sevoflurane)	After induction of anesthesia and before the surgical incision, loading dose of 0.5 μg/kg over 10 min, followed by a maintenance infusion of 0.2 μg/kg/h over the surgery After induction of anesthesia and before the surgical incision, loading dose of 1.0 μg/kg over 10 min, followed by a maintenance infusion of 0.4 μg/kg/h over the surgery	Placebo (normal saline)
Xu et al. (55) (Prospective)	60	3–7 years	Single/China	Vitreoretinal surgery	General anesthesia (sevoflurane)	0.5 μg/kg was administered intravenously over a period of 10 min before induction	Placebo (normal saline)
Ali and Abdellatif (22) (Prospective)	120	2–6 years	Single/Egypt	Adenotonsillectomy	General anesthesia (sevoflurane)	0.3 μg/kg was administered intravenously over 5 min about 5 min before the end of surgery	Propofol 1 mg/kg was administered intravenously over 5 min about 5 min before the end of surgery Placebo (normal saline)
Aydogan et al. (62) (Prospective)	32	12–18 years	Single/Turkey	Scoliosis surgery	General anesthesia (propofol)	0.4 μg/kg/h was administered intravenously to sustain RASS score of −2-+1 after surgery	Midazolam 0.1 mg/kg/h was administered intravenously to sustain RASS score of −2-+1 after surgery
Bhadla et al. (63) (Prospective)	60	5–12 years	Single/India	Ophthalmic day-care surgery	General anesthesia (sevoflurane)	Intravenous 0.4 μg/kg premedication	Midazolam 0.05 mg/kg premedication
Chen et al. (26) (Prospective)	78	2–7 years	Single/China	Strabismus surgery	General anesthesia (sevoflurane)	Loading 1 μg /kg, followed by 1 μg /kg/h infusion after induction of anesthesia	Placebo (normal saline) Ketamine 1 mg/kg intravenously plus 1 mg/kg/h infusion after induction of anesthesia
Gupta et al. (31) (Prospective)	36	8–12 years	Single/India	Corrective surgery spinal dysraphism at lumbosacral area	General anesthesia (sevoflurane)	1 μg/kg bolus over 10 min, followed by 0.5 μg/kg/h as maintenance and discontinued at the beginning of skin closure	Placebo (normal saline)
He et al. (33) (Prospective)	87	3–7 years	Single/China	Elective minor surface surgery	General anesthesia (sevoflurane)	Dexmedetomidine 0.5 μg/kg was administrated after LMA insertion for 10 min Dexmedetomidine 1.0 μg/kg was administrated after LMA insertion for 10 min	Placebo (normal saline)
Kim et al. (37) (Prospective)	40	1–5 years	Single/Korea	Ambulatory hernioplasty or orchiopexy	General anesthesia (sevoflurane)+caudal block	Dexmedetomidine 1 μg/kg was infused, followed by 0.1 μg/kg/h until the end of surgery	Placebo (normal saline)
Hasanin and Sira (75) (Prospective)	80	1–14 years	Single/Egypt	Gastrointestinal endoscopy	Sedation	Loading 2.5 μg/kg was infused over 10 min, followed by 2 μg/kg/h for maintenance	Propofol: loading bolus 2 mg/kg, followed by 100 μg/kg/min for maintenance
Kim et al. (38) (Prospective)	94	1–5 years	Single/Korea	Strabismus surgery	General anesthesia (desflurane)	Continuous infusion with 0.2 μg/kg/h after induction to the end of surgery	Placebo (normal saline)
Sheta et al. (70) (Prospective)	72	3–6 years	Single/ Saudi Arabia	Dental rehabilitation	General anesthesia (sevoflurane)	Intranasal dexmedetomidine 1 μg/kg	Intranasal midazolam 1 μg/kg
Bong et al. (25) (Prospective)	120	2–7 years	Single/Singapore	Magnetic resonance imaging scanning	General anesthesia (sevoflurane)	Intravenous dexmedetomidine 0.3 μg/kg before discontinuation of sevoflurane	Intravenous propofol 1 mg/kg Placebo (normal saline)
Cho et al. (27) (Prospective)	80	1–6 years	Single/Korea	Ambulatory unilateral orchiopexy	General anesthesia (sevoflurane)+caudal block	Dexmedetomidine 1 μg/kg with ropivacaine caudal block	Placebo (normal saline)
Hauber et al. (32) (Prospective)	382	4–10 years	Single/USA	Tonsillectomy with or without adenoidectomy	General anesthesia (sevoflurane)	Dexmedetomidine was administered intravenously at a dose of 0.5 μg/kg /kg over 2 to 3 s at about 5 min before the completion of surgery	Placebo (normal saline)
Jiang et al. (65) (Retrospective)		0–36 months	Single/China	Cardiac surgery	General anesthesia (fentanyl)	0.25–0.75 μg/kg/h from the end of surgery to 1 h of extubation	Midazolam 0.5–3 μg/kg/min from the end of surgery to 1 h of extubation
Lundblad et al. (41) (Prospective)	43	1.5–8 years	Single/Sweden	Outpatient inguinal hernia repair	General anesthesia (sevoflurane)+ ilioinguinal/ iliohypogastric nerve blocks	Ilioinguinal/ iliohypogastric nerve blocks with 0.2% ropivacaine and dexmedetomidine 0.3 μg/kg	Ilioinguinal/ iliohypogastric nerve blocks with 0.2% ropivacaine and placebo (normal saline)
Mukherjee et al. (80) (Prospective)	80	3–7 years	Single/India	Elective day care surgery	General anesthesia (sevoflurane)	1 μg/kg intranasal dexmedetomidine as premedication	4 μg/kg intranasal clonidine as premedication
Peng and Zhang (44) (Prospective)	40	3–24 months	Single/China	Cleft palate repair	General anesthesia (sevoflurane)	Dexmedetomidine 0.8 μg/kg/min was continuously infused after the induction	Placebo (normal saline)
Soliman and Alshehri (52) (Prospective)	150	4–14 years	Single/ Saudi Arabia	Outpatient adenotonsillectomy	General anesthesia (sevoflurane)	An initial loading dose of 0.5 μg/kg (started after induction of anesthesia) over 10 min followed by intravenous infusion 0.1–0.3 μg/kg/h during surgery	Placebo (normal saline)
Yao et al. (56) (Prospective)	89	3–7 years	Single/China	Strabismus surgery	General anesthesia (sevoflurane)	Premedication of intranasal saline or dexmedetomidine 1 μg/kg Premedication of intranasal saline or dexmedetomidine 2 μg/kg	Placebo (normal saline)
Abdelaziz et al. (19) (Prospective)	98	1–7 years	Single/ Saudi Arabia	Strabismus surgery	General anesthesia (sevoflurane)	Intranasal dexmedetomidine(1 μg/kg)	Intranasal midazolam (0.1 mg/kg) Placebo (normal saline)
Ali et al. (23) (Prospective)	90	3–6 years	Single/Egypt	Orthopedic surgeries	General anesthesia (sevoflurane)	Dexmedetomidine 0.3 μg/kg 10 min before the end of surgery.	Ketofol: ketamine 0.25 mg/kg and propofol 1.0 mg/kg in combination 10 min before the end of surgery Placebo (normal saline)
Al-Zaben et al. (21) (Prospective)	75	1–6 years	Single/Jordan	Elective lower abdominal and perineal surgeries	General anesthesia (sevoflurane)+ caudal block	B-D_cau_: 1 ml/kg caudal 0.25% bupivacaine mixed with 1 μg/kg dexmedetomidine B-D_IV_: 1 ml/kg of caudal 0.25% bupivacaine and 1 μg/kg dexmedetomidine and 10 ml intravenously in 0.9% saline over 10 min	B: 1 ml/kg caudal 0.25% bupivacaine and 10 ml 0.9% intravenous saline over 10 min
Eldeek et al. (77) (Prospective)	110	3–7 years	Single/Egypt	Magnetic resonance imaging	Sedation	A loading dose of l μg/kg was given over 10 min, followed by 0.5–0.75 μg/kg/h intravenously	A loading dose of ketamine l mg/kg was given over 10 min, followed by 10–15 μg/kg/min intravenously
Lin et al. (40) (Prospective)	90	1–8 years	Single/China	Cataract surgeries	General anesthesia (sevoflurane)	Intranasally received 1 μg/kg Intranasally received 2 μg/kg	Placebo (normal saline)
Makkar et al. (42) (Prospective)	100	2–8 years	Single/India	Elective infra-umbilical surgery	General anesthesia (desflurane)	0.3 μg/kg intravenous dexmedetomidine over 5 min at 5 min before the end of surgery	A single intravenous bolus of 1 mg/kg propofol at 5 min before the end of surgery Placebo (normal saline)
Song et al. (53) (Prospective)	103	2–6 years	Single/Korea	Strabismus surgery	General anesthesia (sevoflurane + desflurane)	Intravenous 0.25, 0.5, or 1.0 μg/kg for 10 min	Placebo (normal saline)
El-Hamid and Yassin (28) (Prospective)	86	3–7 years	Single/Egypt	Tonsillectomy and/or adenoidectomy	General anesthesia (sevoflurane)	Intranasal dexmedetomidine at 1 μg/kg after induction of general anesthesia	Placebo (normal saline)
Ezz (78) (Prospective)	90	3–6 years	Single/Egypt	Unilateral or bilateral myringotomy	General anesthesia (sevoflurane)	Intranasal dexmedetomidine in a dose 1 μg/kg	Intranasal ketamine in a dose 5 mg/kg
Prabhu and Mehandale (68) (Prospective)	90	1–10 years	Single/India	Elective surgeries of <2 h of expected duration under sevoflurane general anesthesia	General anesthesia (sevoflurane)	Oral dexmedetomidine 4 μg/kg at approximately 45 min before surgery	Oral midazolam 0.5 mg/kg at approximately 45 min before surgery
Keles and Kocaturk (36) (Retrospective)	100	2–6 years	Single/Turkey	Full mouth dental rehabilitation	General anesthesia (sevoflurane)	1 μg/kg oral dexmedetomidine at 45 min before induction of anesthesia	Placebo
Park et al. (73) (Prospective)	57	3–12 years	Single/Korea	Extensive orthopedic surgery of the lower extremities	General anesthesia (sevoflurane)+epidural anesthesia	0.2% ropivacaine (0.2 ml/kg) with dexmedetomidine (1 μg/kg) through the epidural catheter at 30 min before the end of the surgery	0.2% ropivacaine (0.2 ml/kg) with fentanyl (1 μg/kg) through the epidural catheter at 30 min before the end of the surgery
Riveros et al. (46) (Retrospective)	653	0–18 years	Single/USA	Cardiac catheterization	General anesthesia	Received dexmedetomidine infusion during the surgery	Did not receive dexmedetomidine infusion during the surgery
Yuen et al. (81) (Prospective)	196	2–79 months	Multiple/China	Computerized tomographic (CT)	General anesthesia (oral chloral hydrate)	Intranasal dexmedetomidine spray 3 μg/kg, 30 min before computerized tomography studies	Chloral hydrate
Abdel-Ghaffar et al. (60) (Prospective)	90	3–7 years	Single/Egypt	Bone marrow biopsy	General anesthesia (sevoflurane)	Nebulized dexmedetomidine 2 μg/kg as premedication by inhalation	Nebulized ketamine 2 mg/kg as premedication by inhalation Nebulized midazolam 0.2 mg/kg as premedication by inhalation
Li et al. (39) (Prospective)	82	4–6 years	Single/China	Tonsillectomy	General anesthesia (desflurane)	Dexmedetomidine was continuously infused with 0.2 μg/kg/h after anesthesia induction until the end of the surgery	Placebo (normal saline)
Long et al. (66) (Retrospective)	52	3–7 years	Single/Turkey	Full-mouth dental rehabilitation	General anesthesia (sevoflurane)	2 μg/kg of oral dexmedetomidine in apple juice 45 min before the induction of anesthesia	0.5 mg/kg of midazolam in apple juice 45 min before the induction of anesthesia
Tsiotou et al. (54) (Prospective)	60	3–14 years	Single/Greece	Tonsillectomy with or without adenoidectomy	General anesthesia (propofol)	1 μg/kg dexmedetomidine in 10 min after induction	Placebo (normal saline)
Abdel-Ghaffar et al. (20) (Prospective)	90	3–6 years	Single/Egypt	Tonsillectomy	General anesthesia (sevoflurane)	Trans-mucosal dexmedetomidine 0.5 μg/kg Trans-mucosal dexmedetomidine 1 μg/kg	Placebo (normal saline)
Bi et al. (24) (Prospective)	40	6–48 months	Single/China	Tracheobronchial foreign body removal	General anesthesia (sevoflurane)	Intranasal 1 μg/kg at 25 min before anesthesia induction	Placebo (normal saline)
Cho et al. (64) (Prospective)	66	2–12 years	Single/Korea	Tonsillectomy	General anesthesia (sevoflurane)	0.3 μg/kg was administered intravenously for 5 min at 5 min before the end of surgery	Midazolam 0.03 mg/kg was administered intravenously for 5 min at 5 min before the end of surgery
Sajid et al. (69) (Prospective)	80	1–6 years	Single/India	Elective herniotomy	General anesthesia (isoflurane)	Oral dexmedetomidine 4 μg/kg at 40 min before induction	Oral midazolam 0.5 mg/kg at 40 min before induction
Sharma et al. (49) (Prospective)	60	5–10 years	Single/India	Adenotonsillectomy	General anesthesia (isoflurane)	Dexmedetomidine 1 μg/kg infusion over 10 min before induction of anesthesia	Placebo (normal saline)
Shi et al. (50) (Prospective)	90	2–7 years	Single/China	Tonsillectomy	General anesthesia (sevoflurane)	After induction, 0.5 μg/kg over 10 min	Placebo (normal saline)
Ye et al. (57) (Prospective)	60	2–7 years	Single/China	Strabismus and vitreoretinal (VR) surgery	General anesthesia (propofol)	RD: Retrobulbar block with 0.5% ropivacaine 0.1 ml/kg plus dexmedetomidine 1 μg/kg after general anesthesia	RB: Retrobulbar block with 0.5% ropivacaine 0.1 ml/kg only F: General anesthesia alone
Zhang et al. (58) (Prospective)	134	0–16 years	Single/China	Elective interventional cardiac catheterization	General anesthesia (sevoflurane)	An intranasal administration dose of 1.5 mg/kg	Placebo (normal saline)

**Table 2:** The number of patients with EA or ED in dexmedetomidine and control groups.

**Study**	**Dexmedetomidine**		**Control**	
	***N* (total)**	***N* (EA or ED)**	***N* (total)**	***N* (EA or ED)**
Ibacache et al. (34) (Prospective)	0.15 μg/kg: 30 0.3 μg/kg: 30	0.15 μg/kg: 3 0.3 μg/kg: 6	Placebo: 30	4
Guler et al. (30) (Prospective)	30	5	Placebo: 30	17
Shukry et al. (51) (Prospective)	23	6	Placebo: 23	16
Isik et al. (35) (Prospective)	21	1	Placebo: 21	10
Erdil et al. (29) (Prospective)	30	5	Fentanyl: 30 Placebo: 30	Fentanyl: 4 Placebo: 14
Saadawy et al. (47) (Prospective)	30	3	Placebo: 30	9
Talon et al. (71) (Prospective)	50	5	Midazolam: 50	5
Koruk et al. (79) (Prospective)	9	0	Ketamine: 9	1
Patel et al. (74) (Prospective)	61	11	Fentanyl: 61	25
Sato et al. (48) (Prospective)	39	11	Placebo: 42	27
Bedirli et al. (72) (Prospective)	38	4	Tramadol: 39	5
Mason et al. (76) (Retrospective)	1274	4	Pentobarbital: 388	8
Mountain et al. (67) (Prospective)	22	3	Midazolam: 19	5
Özcengiz et al. (59) (Prospective)	25	2	Midazolam: 25 Melatonin: 25 Placebo: 25	Midazolam: 1 Melatonin: 2 Placebo: 8
Pestieau et al. (45) (Prospective)	1 μg/kg: 23 2 μg/kg: 28	1 μg/kg: 5 2 μg/kg: 9	Fentanyl: 23 Placebo: 27	Fentanyl: 3 Placebo: 11
Akin et al. (61) (Prospective)	45	8	Midazolam: 45	5
Meng et al. (43) (Prospective)	0.5 μg/kg: 40 1.0 μg/kg: 40	0.5 μg/kg: 6 1.0 μg/kg: 2	Placebo: 40	8
Xu et al. (55) (Prospective)	30	3	Placebo: 30	13
Ali and Abdellatif (22) (Prospective)	40	2	Propofol: 40 Placebo: 40	Propofol: 3 Placebo: 7
Aydogan et al. (62) (Prospective)	16	1	Midazolam: 16	4
Bhadla et al. (63) (Prospective)	30	7	Midazolam: 30	14
Chen et al. (26) (Prospective)	27	3	Placebo: 24 Ketamine: 27	Placebo: 11 Kertamine: 6
Gupta et al. (31) (Prospective)	18	0	Placebo: 18	4
He et al. (33) (Prospective)	0.5 μg/kg: 29 1 μg/kg: 32	0.5 μg/kg: 5 1 μg/kg: 2	Placebo: 26	11
Kim and Koo (37) (Prospective)	20	1	Placebo: 20	11
Hasanin and Sira (75) (Prospective)	40	0	Propofol: 40	0
Kim et al. (38) (Prospective)	47	7	Placebo: 47	33
Sheta et al. (70) (Prospective)	36	4	Midazolam: 36	11
Bong et al. (25) (Prospective)	40	3	Propofol: 39 Placebo: 41	Propofol: 0 Placebo: 2
Cho et al. (27) (Prospective)	40	3	Placebo: 40	18
Hauber et al. (32) (Prospective)	193	69	Placebo: 189	125
Jiang et al. (65) (Retrospective)	77	14	Midazolam: 97	31
Lundblad et al. (41) (Prospective)	22	0	Placebo: 21	4
Mukherjee et al. (80) (Prospective)	40	9	Clonidine: 40	14
Peng and Zhang (44) (Prospective)	20	3	Placebo: 20	18
Soliman et al. (52) (Prospective)	75	8	Placebo: 75	23
Yao et al. (56) (Prospective)	1 μg/kg: 30 2 μg/kg: 30	1 μg/kg: 5 2 μg/kg: 1	Placebo: 29	14
Abdelaziz et al. (19) (Prospective)	33	4	Placebo: 32 Midazolam: 33	Placebo: 15 Midazolam: 7
Ali et al. (23) (Prospective)	30	EA: 5	Ketofol: 30 Placebo: 30	Ketofol: 8 Placebo: 27
Al-Zaben et al. (21) (Prospective)	B-D_cau_: 25 B-D_IV_: 25	B-D_cau_: 0 B-D_IV_: 2	B: 25	8
Eldeek et al. (77) (Prospective)	55	0	Ketamine: 55	2
Lin et al. (40) (Prospective)	1 μg/kg: 30 2 μg/kg: 30	1 μg/kg: 7 2 μg/kg: 3	Placebo: 30	24
Makkar et al. (41) (Prospective)	32	3	Propofol: 36 Placebo: 32	Propofol: 5 Placebo: 13
Song et al. (53) (Prospective)	0.25 μg/kg: 25 0.5 μg/kg: 25 μg/kg: 28	0.25 μg/kg: 12 0.5 μg/kg: 11 1.0 μg/kg: 6	Placebo: 28	15
El-Hamid and Yassin (28) (Prospective)	43	3	Placebo: 43	25
Ezz (78) (Prospective)	45	3	Ketamine: 45	3
Prabhu and Mehandale (68) (Prospective)	45	2	Midazolam: 45	18
Keles and Kocaturk (36) (Retrospective)	50	6	Placebo: 50	12
Park et al. (73) (Prospective)	28	5	Fentanyl: 29	8
Riveros et al. (46) (Retrospective)	331	48	Placebo: 322	44
Yuen et al. (81) (Prospective)	87	4	Chloral hydrate: 107	5
Abdel-Ghaffar et al. (60) (Prospective)	30	2	Ketamine: 30 Midazolam: 30	Ketamine: 6 Midazolam: 12
Li et al. (38) (Prospective)	40	6	Placebo: 40	33
Long et al. (66) (Retrospective)	26	0	Midazolam: 26	5
Tsiotou et al. (54) (Prospective)	31	15	Placebo: 29	6
Abdel-Ghaffar et al. (20) (Prospective)	0.5 μg/kg: 30 1 μg/kg: 30	0.5 μg/kg: 18 1 μg/kg: 16	Placebo: 30	15
Bi et al. (24) (Prospective)	20	5	Placebo: 20	14
Cho et al. (24) (Prospective)	34	9	Midazolam: 32	10
Sajid et al. (69) (Prospective)	40	9	Midazolam: 40	32
Sharma et al. (49) (Prospective)	30	2	Placebo: 30	30
Shi et al. (50) (Prospective)	45	14	Placebo: 45	24
Ye et al. (57) (Prospective)	RD: 20	4	RB: 20 F: 20	RB: 7 F: 17
Zhang et al. (83) (Prospective)	67	6	Placebo: 67	13

### Bias Risk Assessment

Bias risk of 58 RCTs was assessed by the Cochrane Collaboration Risk of Bias Assessment tool. Random sequence generation was assessed as a low risk of bias in 57 studies (98%), allocation concealment was assessed in 36 studies (62%), blinding of participants was assessed in 38 studies (66%), blinding of outcome assessment was assessed in 34 studies (59%), incomplete outcome data were assessed in 58 studies (100%), and selective outcome reporting was assessed in 56 studies (97%). Nineteen RCTs (24, 26, 27, 32, 33, 37, 41, 45, 53, 54, 56, 58–61, 64, 67, 73, 81) were assessed to be of high quality (Supplementary Figures 1, 2). Bias risk of 5 CCTs (36, 46, 65, 66, 76) was assessed by NOS, and the number of stars was 7 from the study of Keles et al. (36), 8 from the study of Riveros et al. (46), 5 from the study of Jiang et al. (65), 5 from the study of Long et al. (66), and 8 from the study of Mason et al. (76), respectively. Therefore, 3 trials (36, 46, 76) were assessed to be of high quality because they obtained 7 stars or more (Supplementary Table 3).

### Post-anesthesia Incidence of EA or ED

Different dosages of dexmedetomidine administration in each study were presented in nine trials (20, 21, 33, 34, 40, 43, 45, 53, 56). We chose the dexmedetomidine dosage with the highest incidence of EA or ED. We evaluated the effect of dexmedetomidine administration on EA or ED compared with placebo (19–59), midazolam (19, 59–71), opioids (29, 45, 72–74), propofol (or pentobarbital) (22, 25, 42, 75, 76), ketamine (26, 60, 77–79), and other sedatives (clonidine, chloral hydrate, melatonin) or ketofol (23, 59, 80, 81).

The random-effect model with OR was selected due to high *I*^2^ in the groups of placebo (*I*^2^ = 75%), midazolam (*I*^2^ = 57%), and propofol (or pentobarbital) (*I*^2^ = 58%), whereas the fixed-effect model with OR was selected because of low I^2^ in the group of opioids (*I*^2^ = 0%) and ketamine (*I*^2^ = 0%).

**Figure 2:**
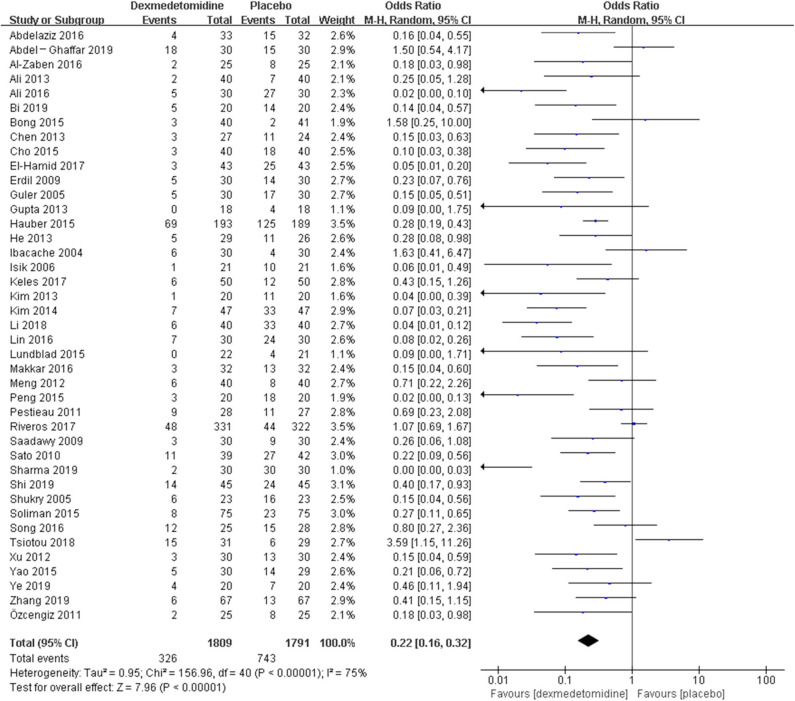
Comparison of pediatric EA or ED between dexmedetomidine and placebo groups.

**Figure 3:**
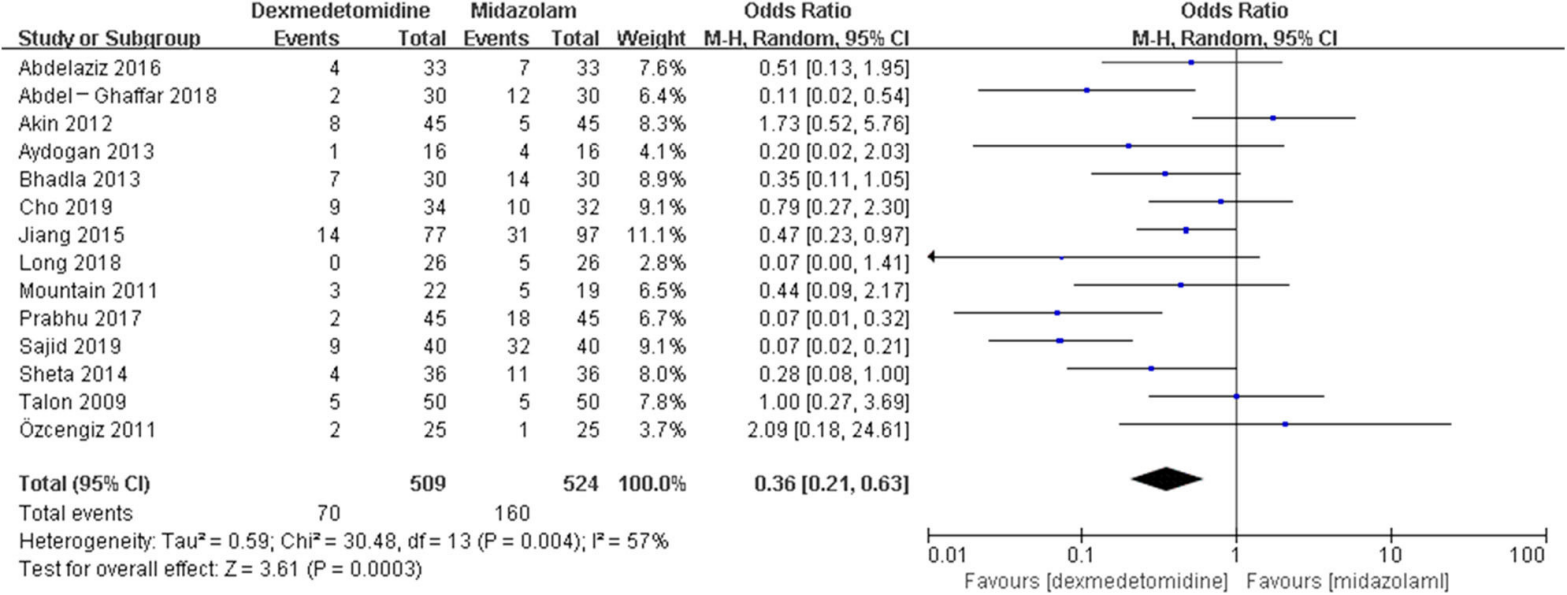
Comparison of pediatric EA or ED between dexmedetomidine and midazolam groups.

**Figure 4:**
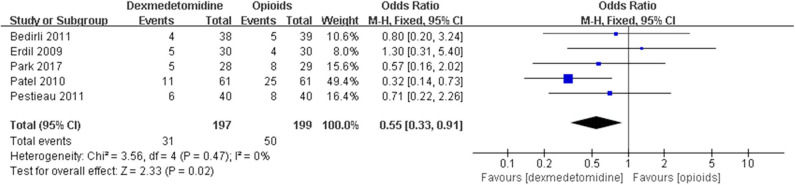
Comparison of pediatric EA or ED between dexmedetomidine and opioids groups.

**Figure 5:**
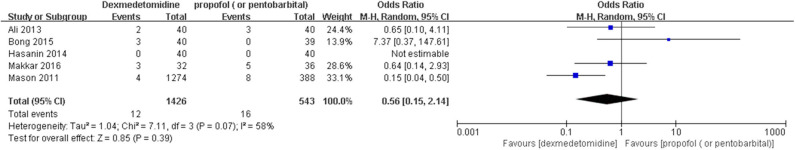
Comparison of pediatric EA or ED between dexmedetomidine and propofol (or pentobarbital) groups.

**Figure 6:**
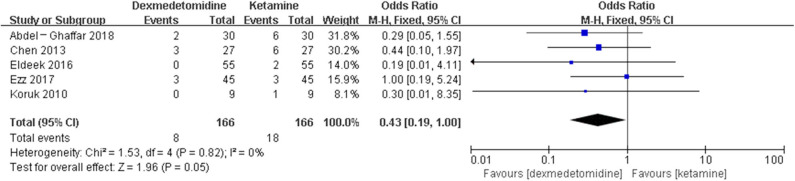
Comparison of pediatric EA or ED between dexmedetomidine and ketamine groups.

The pooled results demonstrated significant difference in the incidence of EA or ED after anesthesia in the groups of placebo [OR = 0.22, 95% CI: (0.16, 0.32), *I*^2^ = 75%, *P* for effect <0.00001] (Figure 2), midazolam [OR = 0.36, 95% CI: (0.21, 0.63), *I*^2^ = 57%, *P* for effect = 0.0003] (Figure 3), and opioids [OR = 0.55, 95% CI: (0.33, 0.91), *I*^2^ = 0, *P* for effect = 0.02] (Figure 4). However, no significant difference was exhibited in the groups of propofol (or pentobarbital) [OR = 0.56, 95% CI: (0.15, 2.14), *I*^2^ = 58%, *P* for effect = 0.39] (Figure 5) and ketamine [OR = 0.43, 95% CI: (0.19, 1.00), *I*^2^ = 0, *P* for effect = 0.05] (Figure 6).

With regard to other control sedatives or drug combination, no heterogenicity was presented because only one literature was retrieved for each group. The results did not demonstrate significant difference in the incidence of EA or ED after anesthesia when comparing dexmedetomidine with clonidine [OR = 0.54, 95% CI: (0.20, 1.45), *P* for effect = 0.22], chloral hydrate [OR = 0.98, 95% CI: (0.26, 3.78), *P* for effect = 0.98], melatonin [OR = 1.0, 95% CI: (0.13, 7.72), *P* for effect = 1.00], and ketofol [OR = 0.55, 95% CI: (0.16, 1.93), *P* for effect = 0.35].

### Sensitivity Analysis

Meta-regression was performed to investigate the heterogeneity sources by assessing the potential factors including the year of publication, study methods, the country of authors, the time of drug administration, the type of surgery, routes of drug administration, the bias risk of the study, and the range of patients' age for the groups of placebo and midazolam. Unexpectedly, all *P*-values of these risk factors were over 0.05 (Supplementary Tables 4, 5). Afterward, the method of one-by-one literature removal was used. Seven trials (20, 23, 39, 44, 46, 49, 54) were found to be the main sources of heterogeneity in the placebo group (*I*^2^ dropped from 75 to 36%) and two trials (61, 69) in the midazolam group (*I*^2^ dropped from 57 to 28%). Due to a small number of included trials in the group of propofol (or pentobarbital), the method of one-by-one literature removal was directly used to lower the heterogeneity. When we removed the retrospective trial from Mason et al. (76), the value of *I*^2^ in the propofol (or pentobarbital) group dropped from 58 to 13%, and the changes suggested that this retrospective trial was the main source of significant heterogeneity.

**Figure 7:**
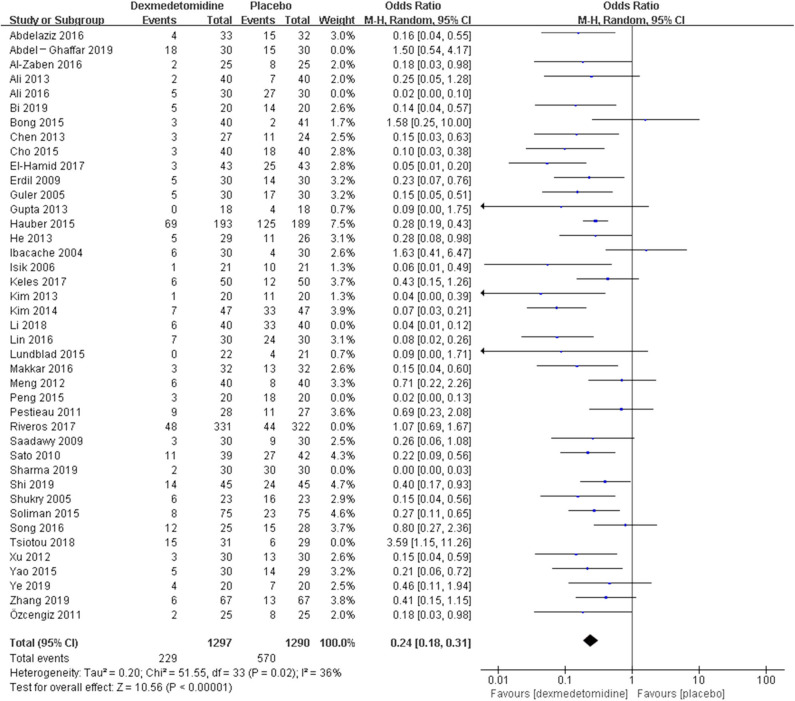
Comparison of pediatric EA or ED between dexmedetomidine and placebo groups after sensitivity analysis.

**Figure 8:**
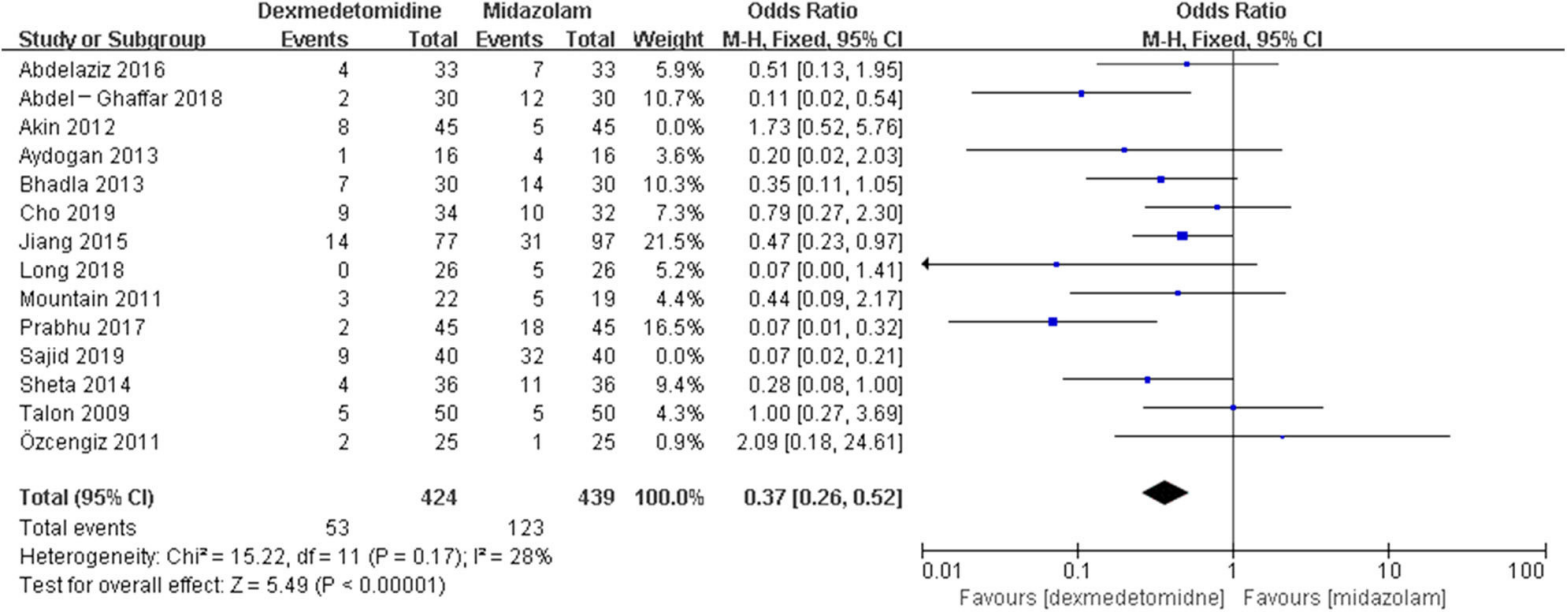
Comparison of pediatric EA or ED between dexmedetomidine and midazolam groups after sensitivity analysis.

**Figure 9:**
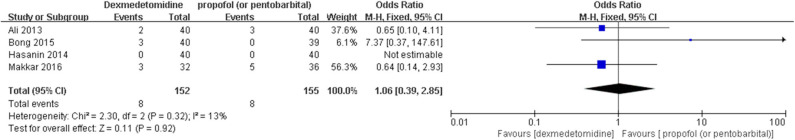
Comparison of pediatric EA or ED between dexmedetomidine and propofol (or pentobarbital) groups after sensitivity analysis.

The *post hoc* analysis was performed by the fixed-effects model with OR, and the pooled results were consistent with those prior to the sensitivity analysis—placebo group: [OR = 0.24, 95% CI: (0.18, 0.31), *I*^2^ = 36%, *P* for effect <0.00001] (Figure 7); midazolam group: [OR = 0.37, 95% CI: (0.26, 0.52), *I*^2^ = 28%, *P* for effect <0.00001] (Figure 8); propofol (or pentobarbital) group: [OR = 1.06, 95% CI: (0.39, 2.85), *I*^2^ = 13%, *P* for effect = 0.92] (Figure 9).

## Discussion

This meta-analysis included 58 RCTs and 5 CCTs that compared the prophylactic effect of dexmedetomidine vs. placebo or other sedatives on post-anesthesia EA or ED in pediatric patients undergoing medical procedures. The results showed that dexmedetomidine strikingly decreased the incidence of post-anesthesia EA or ED compared with placebo, midazolam, or opioids, whereas the significant difference was not exhibited compared with propofol (or pentobarbital), ketamine, clonidine, chloral hydrate, melatonin, and ketofol, respectively.

Currently, the specific predisposing causes of EA or ED following medical procedures in children remain unclear. Children undergoing general anesthesia are prone to suffer post-anesthesia EA or ED due to their immature central nervous system, preoperative fear and anxiety about unfamiliar surroundings, and postoperative pain (84–86). In addition, the children undergoing inhalation anesthesia through sevoflurane, isoflurane, or desflurane may suffer from a high incidence of post-anesthesia EA or ED (87, 88). Various medications have been used to prevent EA or ED in pediatric patients, like benzodiazepines, opioids, propofol, ketamine, clonidine, dexmedetomidine, and so on (11, 89–93).

Dexmedetomidine, as a highly selective α_2_ adrenergic receptor agonist, can produce pharmacological effects of anti-anxiety, sedation, and analgesia without overt respiratory and circulatory inhibition in a routine dose (94, 95). Meanwhile, dexmedetomidine can improve the cognitive function in children during recovery from general anesthesia (96) and contributes to dose-dependent inhibition of EA or ED after medical procedures (97). The optimal dose (ED_95_) of dexmedetomidine for preventing EA was 0.30 μg/kg (95% CI: 0.21–1.00 μg/kg) (83). An animal experiment demonstrated that dexmedetomidine could enhance spatial learning and memory in neonatal rats under physiological conditions through promoting hippocampal neurogenesis (98). In this meta-analysis, nine trials had different dexmedetomidine groups according to different dosages (20, 33, 34, 40, 43, 45, 53, 56) or administration routes of this drug (21). Patients in the control groups of these nine trials were treated with a placebo (20, 21, 33, 34, 40, 43, 45, 53, 56), and patients in another control group in the study from Pestieau et al. received fentanyl treatment (45). We chose the dexmedetomidine group with higher incidence of EA or ED. Therefore, the pooled results were more convincing in the powerful prophylactic effect of dexmedetomidine on the occurrence of EA or ED in children compared with placebo and opioids.

Dexmedetomidine can be administered in a variety of ways, like intravenous, transnasal, oral, inhalation, caudal or nerve block, and so on; thus, pediatric patients can easily accept it. The pooled results of 53 trials comparing dexmedetomidine with placebo and midazolam showed that dexmedetomidine could work in various ways and was superior to placebo or midazolam in inhibiting EA or ED in children. However, compared with propofol (or pentobarbital) or ketamine, dexmedetomidine did not demonstrate its superiority in reducing pediatric EA or ED following anesthesia. The possible explanations included the following: (1) the efficacy of propofol (or pentobarbital) or ketamine in suppressing EA or ED occurrence was no less than that of dexmedetomidine; and (2) the number of relevant prospective studies needed to be further increased. Because only one article was included, we could not perform meta-analysis for trials in the group of clonidine, chloral hydrate, melatonin, or ketofol.

In this meta-analysis, high heterogenicity was detected in trials comparing dexmedetomidine with placebo (*I*^2^ = 75%), midazolam (*I*^2^ = 57%), and propofol (or pentobarbital) (*I*^2^ = 58%), respectively. Subgroup analysis is an effective method to solve large heterogenicity among studies (99). We suggested some possible risk factors associated with overt heterogenicity including the year of publication, study methods, the country of authors, the time of drug administration, the type of surgery, routes of drug administration, the bias risk of the study, and the range of patients' age. Meta-regression was used to identify heterogenicity sources. If the *P*-value of meta-regression was <0.05 through analyzing one risk factor, the subgroup analysis was performed based on this risk factor (99, 100). However, in this meta-analysis, all *P*-values of meta-regression were more than 0.05 through analyzing all possible risk factors in the placebo and midazolam groups. Hence, we considered that significant heterogeneity may be the result of a combination of multiple factors. The meta-analysis by a random-effect model can decrease the effect of significant heterogeneity on the results, although this method does not solve heterogeneity (101). In addition, the method of trial exclusion is also an effective method to solve large heterogenicity for meta-analysis (102). When we excluded seven trials (20, 23, 39, 44, 46, 49, 54) in the placebo group, two trials (61, 69) in the midazolam group, and one trial (76) in the propofol (or pentobarbital) group, all values of *I*^2^ dropped to below 40%. Interestingly, the pooled results were consistent with those prior to sensitivity analysis.

It is necessary to elaborate the strengths and limitations of our meta-analysis. Firstly, this meta-analysis presented a comprehensive and up-to-date analysis of dexmedetomidine vs. placebo or other sedatives in pediatric patients. Sixty-three included trials with unlimited study methods (RCTs and CCTs) and various administration routes and dosages were grouped according to control drugs; thus, the pooled outcomes revealed the effect of dexmedetomidine on pediatric EA or ED more comprehensively. Secondly, sensitivity analysis was conducted in groups with high heterogeneity to remove the influence of heterogeneity on the overall results. Thirdly, this meta-analysis provided several directions for future clinical studies about the effect of dexmedetomidine on EA or ED in children. In addition, some limitations should be taken into account in this meta-analysis. Foremost, 39 RCTs and 2 CCTs in 63 included trials were assessed to be high bias risk, and so many trials with high-risk bias would affect the results. Additionally, the age gap of participants in 9 trials (46, 52, 54, 58, 64, 71, 72, 75, 79) was over 10 years, and a large age gap might be an important risk factor associated with the unreliability of outcomes. Lastly, non-uniform definitions of EA or ED were an additional limitation of this meta-analysis. There were five strategies diagnosing EA or ED in included trials, i.e., three-point scale, four-point scale, five-point scale, pediatric Anesthesia Emergence Delirium (PAED) scale, and the Confusion Assessment Method for the ICU.

## Conclusion

In conclusion, compared with placebo, midazolam, and opioids, dexmedetomidine significantly decreased the incidence of post-anesthesia EA or ED in pediatric patients. However, dexmedetomidine did not exhibit this superiority when compared with propofol and ketamine. With regard to clonidine, chloral hydrate, melatonin, or ketofol, the results needed to be further tested due to the fact that there was only one trial in each study.

## Data Availability Statement

All datasets presented in this study are included in the article/Supplementary Material.

## Author Contributions

XW and JL designed this meta-analysis and supervised the acquisition and analysis of the data. YR, RZ, and XJ were independently responsible for reviewing the titles, abstracts, or both and summarized the data of the included literatures. RZ and XJ conducted statistical analysis of the data. YR wrote the manuscript. All authors contributed to the article and approved the submitted version.

## Conflict of Interest

The authors declare that the research was conducted in the absence of any commercial or financial relationships that could be construed as a potential conflict of interest.

## Supplementary Material

Supplementary Figure 1 Risk of bias graph: review authors' judgments about each risk of bias item presented as percentages across all included studies.

Supplementary Figure 2 Risk of bias summary: review authors' judgments about each risk of bias item for each included study.

Supplementary Table 1 The guidelines of the 2009 PRISMA (Preferred Reporting Items for Systematic reviews and Meta-analyses).

Supplementary Table 2 The Newcastle-Otawa Quality Assessment Scale (NOS).

Supplementary Table 3 The bias risk of CCTs by Newcastle-Otawa Quality Assessment Scale (NOS).

Supplementary Table 4 Meta-regression (dexmedetomidine vs. placebo): estimate of between-study variance % residual variation due to heterogeneity.

Supplementary Table 5 Meta-regression (dexmedetomidine vs. midazolam): estimate of between-study variance % residual variation due to heterogeneity.
