# Supplementary figures and images for: The Effect of Dexmedetomidine on Emergence Agitation or Delirium in Children After Anesthesia—A Systematic Review and Meta-Analysis of Clinical Studies
Source: Front Pediatr. 2020 Jul 14;8:329. doi: 10.3389/fped.2020.00329 (PMC7381209; doi:10.3389/fped.2020.00329)

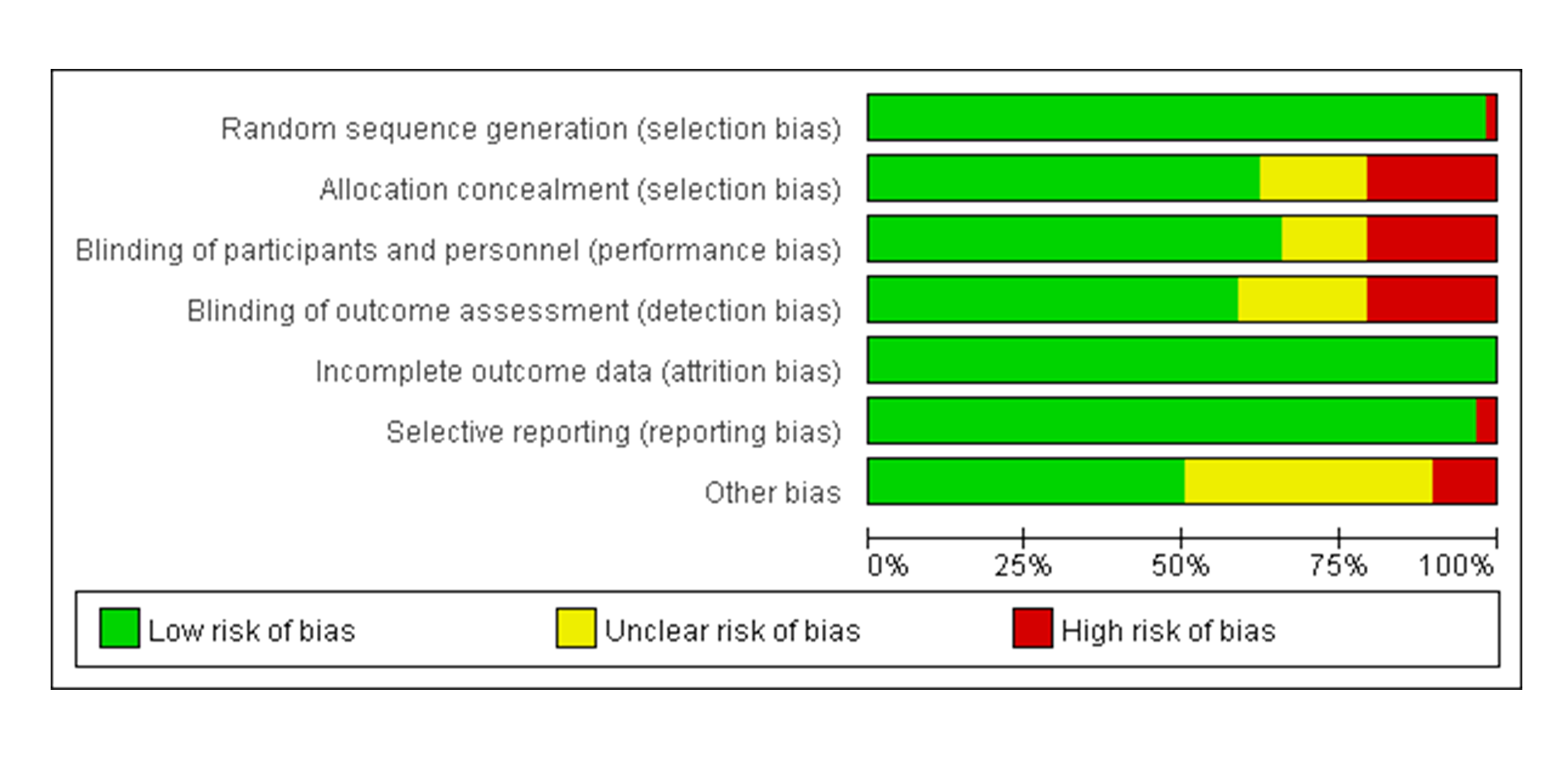

Supplement: Supplementary Figure 1 — Risk of bias graph: review authors' judgments about each risk of bias item presented as percentages across all included studies. [file Image_1.TIF]

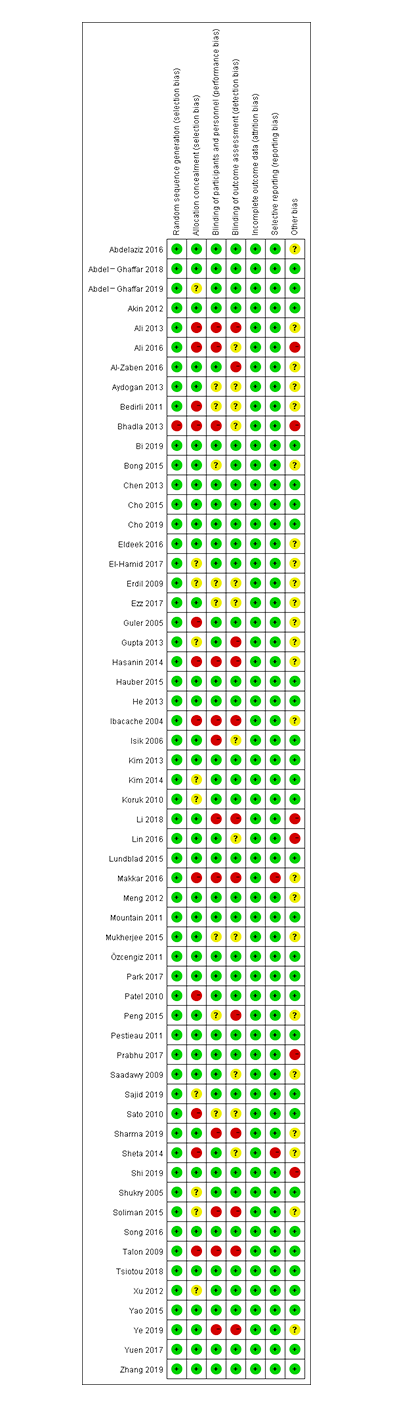

Supplement: Supplementary Figure 2 — Risk of bias summary: review authors' judgments about each risk of bias item for each included study. [file Image_2.TIF]
