# Supplementary material for: The Effect of Dexmedetomidine on Emergence Agitation or Delirium in Children After Anesthesia—A Systematic Review and Meta-Analysis of Clinical Studies
Source: Front Pediatr. 2020 Jul 14;8:329. doi: 10.3389/fped.2020.00329 (PMC7381209; doi:10.3389/fped.2020.00329)
Supplement: Supplementary Table 1 — The guidelines of the 2009 PRISMA (Preferred Reporting Items for Systematic reviews and Meta-analyses). [file Table_1.DOC]

| **Section/topic** | **#** | **Checklist item** | **Reported on page #** |
| --- | --- | --- | --- |
| **TITLE** | | |  |
| Title | 1 | The effect of dexmedetomidine on emergence agitation or delirium in children after anesthesia---a systematic review and meta-analysis of clinical studies | P 1 |
| **ABSTRACT** | | |  |
| Structured summary | 2 | **Background:** We conducted this systematic review and meta-analysis to investigate the clinical effect of dexmedetomidine in preventing pediatric emergence agitation (EA) or delirium (ED) following anaesthesia compared with placebo or other sedatives.  **Methods:** The databases of Pubmed, Embase and Cochrane Library were searched till 8th January 2020. Inclusion criteria were participants with age<18 yr and studies of comparison between dexmedetomidine and placebo or other sedatives. Exclusion criteria included adult studies, duplicate publications, management with dexmedetomidine alone, review or meta-analysis, basic research, article published as abstract, letter, case report, editorial, note, method or protocol, and article presented in non-English language.  **Results:** 58 Randomized Controlled Trials (RCTs) and 5 Case-control Trials (CCTs) including 7714 patients were included. The results showed that dexmedetomidine significantly decreased the incidence of post-anesthesia EA or ED compared with placebo [OR=0.22, 95% CI: (0.16, 0.32), I^2^=75, *P* < 0.00001], midazolam [OR=0.36, 95% CI: (0.21, 0.63), I^2^=57, *P* =0.0003], and opioids [OR=0.55, 95% CI: (0.33, 0.91), I^2^=0, *P* =0.02], whereas the significant difference was not exhibited compared with propofol (or pentobarbital) [OR=0.56, 95% CI: (0.15, 2.14), I^2^=58, *P* =0.39], ketamine [OR=0.43, 95% CI: (0.19, 1.00), I^2^=0, *P* =0.05], clonidine [OR=0.54, 95% CI: (0.20, 1.45), *P* =0.22], chloral hydrate [OR=0.98, 95% CI: (0.26, 3.78), *P* =0.98], melatonin [OR=1.0, 95% CI: (0.13, 7.72), *P* =1.00], and ketofol [OR=0.55, 95% CI: (0.16, 1.93), *P* =0.35].  **Conclusion:** Compared with placebo, midazolam and opioids, dexmedetomidine significantly decreased the incidence of post-anesthesia EA or ED in pediatric patients. However, dexmedetomidine did not exhibit this superiority compared with propofol and ketamine. With regard to clonidine, chloral hydrate, melatonin and ketofol, the results needed to be further testified due to only one included trial for each control drug.  **Keywords: d**exmedetomidine, pediatric, agitation, delirium, meta-analysis | P 2 |
| **INTRODUCTION** | | |  |
| Rationale | 3 | Emergence agitation (EA) or delirium (ED) manifests as a series of sudden complex psychomotor disorders, charaterized by perceptual disturbances, delusions, and disorientation following sedation or general anesthesia. The incidence of EA or ED after general anesthesia in children ranges from 10% to 80%, and significantly increases the occurrence of other complications after anesthesia, like self-injury, prolonged post-anesthesia care unit (PACU) stay, poor satisfication of parents and care providers and so on. Activation of α_2_ adrenergic receptor can contribute to pharmacological effects of sedation, analgesia and anti-inflammation, thus α_2_ adrenergic receptor may be a target for prevention and treatment of EA or ED. Although dexmedetomidine is used as an off-label drug in children, increasing studies about effect of dexmedetomidine on EA and ED in pediatric patients have been completed. | P 3 |
| Objectives | 4 | We conducted this meta-analysis for clinical trials to evaluate the effect of dexmedetomidine on EA or ED following sedation or general anesthesia in pediatric patients compared with placebo and other drugs. |  |
| **METHODS** | | |  |
| Protocol and registration | 5 | No registration |  |
| Eligibility criteria | 6 | The inclusion criteria included: 1) participants with age<18 yr; 2) management with prophylactic dexmedetomidine and placebo or other sedatives. The exclusion criteria included: 1) participants with age≥18 yr; 2) management with dexmedetomidine alone; 3) review or meta-analysis; 4) basic research; 5) article published as abstract, letter, case report, editorial, note, method or protocol; 6) article presented in non-English language. | P 4 |
| Information sources | 7 | We searched the databases including "Pubmed", "Embase" and "Cochrane Library" through PICOS (Population, Intervention, Comparison, Outcome, Study design) method by the time to 8th January 2020. | P 3-4 |
| Search | 8 | The entry words included "child" OR "children" OR "pediatric" AND "dexmedetomidine" OR "precedex" OR "MPV-1440" OR "MPV 1440" AND "agitation" OR "delirium", and the search scope was "all fields". Because all studies about effect of dexmedetomidine versus other drugs (placebo or other sedatives) on agitation or delirium in pediatric patients were eligible in this meta-analysis, we did not confine the search words of control drugs and study design. |  |
| Study selection | 9 | The inclusion criteria included: 1) participants with age<18 yr; 2) management with prophylactic dexmedetomidine and placebo or other sedatives. The exclusion criteria included: 1) participants with age≥18 yr; 2) management with dexmedetomidine alone; 3) review or meta-analysis; 4) basic research; 5) article published as abstract, letter, case report, editorial, note, method or protocol; 6) article presented in non-English language. | P 4 |
| Data collection process | 10 | Three authors were independently responsible for reviewing the titles, abstracts or both and summarized the data of the included literatures. Another two authors were in charge of the data discrepancy adjustment. | P 4-5 |
| Data items | 11 | 1) authors; 2) publication year; 3) number of the total participants in each study; 4) age range of all the participants; 5) country of publication; 6) procedures that the participants underwent 7) time of dexmedetomidine or other sedatives administration; 8) infusion speed or doseage of dexmedetomidine or other sedatives; 9) number of patients with EA or ED following sedation or general anesthesia. | P 4 |
| Risk of bias in individual studies | 12 | The Cochrane Collaboration Risk of Bias Assessment tool was used to assess the risk of bias of all included RCTs, and the Newcastle-Otawa Quality Assessment Scale (NOS) was used to assess the bias risk of case-control trials (CCTs) were assessed by two authors independently. If the two authors had the different assessment results, they consulted the third or the forth one. Eventually, we reached consensus. | P 4-5 |
| Summary measures | 13 | The dichotomous outcome were reported as odds ratios (OR) with 95% confidence interval (CI). The statistical tests were two-sided and *P* value for overall effect <0.05 was considered significant differences. | P 5 |
| Synthesis of results | 14 | The values of I^2^ and the Mantel-Haenszel chi-square test (*P* value for heterogeneity) were used to evaluate the heterogeneity of included studies. And the values of I^2^<40%, 40%-60%, and >60% represented low, moderate and high heterogeneity, respectively. A *P* value for heterogeneity<0.1 or I^2^ >50% was regarded as high heterogeneity and the method of random-effect model analysis was applied to pool the data. | P 5 |

Page 1 of 2

| **Section/topic** | **#** | **Checklist item** | **Reported on page #** |
| --- | --- | --- | --- |
| Risk of bias across studies | 15 | Two authors evaluated the quality and bias risk of the included studies through Cochrane Review Handbook dependently. Bias risk of 58 RCTs was assessed by the Cochrane Collaboration Risk of Bias Assessment tool. Random sequence generation was assessed as a low risk of bias in 57 studies (98%), allocation concealment was assessed in 36 studies (62%), blinding of participants was assessed in 38 studies (66%), blinding of outcome assessment was assessed in 34 studies (59%), incomplete outcome data was assessed in 58 studies (100%) and selective outcome reporting was assessed in 56 studies (97%). 19 RCTs were assessed to be high quality. Bias risk of 5 CCTs was assessed by NOS, and the number of stars were 7 from study of Keles et al., 8 from study of Riveros et al., 5 from study of Jiang et al., 5 from study of Long et al., 8 from study of Mason et al., respectively. Therefore, 3 trials were assessed to be high quality because they obtained 7 stars or more. | P 6-7 |
| Additional analyses | 16 | Meta-regression was performed to investigate the heterogeneity sources by assessing the potential factors including year of publication, study methods, country of authors, time of drug administration, type of surgery, routes of drug administration, bias risk of study, and range of patients' age for the groups of placebo and midazolam. All *P* values of these risk factors were over 0.05 | P 5 |
| **RESULTS** | | |  |
| Study selection | 17 | See Fig. 1 | P 5-6 |
| Study characteristics | 18 | For each study, present characteristics for which data were extracted (e.g., study size, PICOS, follow-up period) and provide the citations. | P 6 |
| Risk of bias within studies | 19 | Present data on risk of bias of each study and, if available, any outcome level assessment (see item 12). | P 6-7 |
| Results of individual studies | 20 | For all outcomes considered (benefits or harms), present, for each study: (a) simple summary data for each intervention group (b) effect estimates and confidence intervals, ideally with a forest plot. | P 7 |
| Synthesis of results | 21 | The random-effect model with OR was selected due to high I^2^ in group of placebo (I^2^ =75%), midazolam (I^2^ =57%), and propofol (or pentobarbital) (I^2^ =58%), whereas the fixed-effect model with OR was selected because of low I^2^ in group of opioids (I^2^ =0%) and ketamine (I^2^ =0%). The pooled results demonstrated significant difference in incidence of EA or ED after anesthesia in group of placebo [OR=0.22, 95% CI: (0.16, 0.32), I^2^=75%, *P* for effect < 0.00001], midazolam [OR=0.36, 95% CI: (0.21, 0.63), I^2^=57%, *P* for effect =0.0003], and opioids [OR=0.55, 95% CI: (0.33, 0.91), I^2^=0, *P* for effect =0.02]. However, no significant difference was exhibited in group of propofol (or pentobarbital) [OR=0.56, 95% CI: (0.15, 2.14), I^2^=58%, *P* for effect =0.39] and ketamine [OR=0.43, 95% CI: (0.19, 1.00), I^2^=0, *P* for effect =0.05]. With regard to other control sedatives or drug combination, no heterogenicity was presented because only one literature was retrieved for each group. The results did not demonstrate significant difference in incidence of EA or ED after anesthesia compared dexmedetomidine with clonidine [OR=0.54, 95% CI: (0.20, 1.45), *P* for effect =0.22], chloral hydrate [OR=0.98, 95% CI: (0.26, 3.78), *P* for effect =0.98], melatonin [OR=1.0, 95% CI: (0.13, 7.72), *P* for effect =1.00], and ketofol [OR=0.55, 95% CI: (0.16, 1.93), *P* for effect =0.35]. | P 7 |
| Risk of bias across studies | 22 | Present results of any assessment of risk of bias across studies (see Item 15). | P 6-7 |
| Additional analysis | 23 | Give results of additional analyses, if done (e.g., sensitivity or subgroup analyses, meta-regression [see Item 16]). | P 8 |
| **DISCUSSION** | | |  |
| Summary of evidence | 24 | This meta-analysis included 58 RCTs and 5 CCTs that compared the prophylactic effect of dexmedetomidine versus placebo or other sedatives on post-anesthesia EA or ED in pediatric patients undergoing medical procedures. The results showed that dexmedetomidine strikingly decreased the incidence of post-anesthesia EA or ED compared with placebo, midazolam, or opioids, whereas the significant difference was not exhibited compared with propofol (or pentobarbital), ketamine, clonidine, chloral hydrate, melatonin and ketofol, respectively. | P 8 |
| Limitations | 25 | Foremost, 39 RCTs and 2 CCTs in 63 included trials were assessed to be high bias risk, and so many trials with high-risk bias would affect the results. Additionally, the age gap of participants in 9 trials was over 10 years and large age gap might be an important risk factors associated with unreliability of outcomes. Lastly, non-uniform definitions of EA or ED were an additional limitation of this meta-analysis. There were five strategies diagnosing EA or ED in included trials, i.e., 3-point scale, 4-point scale, 5-point scale, pediatric Anesthesia Emergence Delirium (PAED) scale, the Confusion Assessment Method for the ICU. | P 10-11 |
| Conclusions | 26 | In conclusion, compared with placebo, midazolam and opioids, dexmedetomidine significantly decreased the incidence of post-anesthesia EA or ED in pediatric patients. However, dexmedetomidine did not exhibit this superiority compared with propofol and ketamine. With regard to clonidine, chloral hydrate, melatonin or ketofol, the results needed to be further testified due to only one trial in each study. | P 11 |
| **FUNDING** | | |  |
| Funding | 27 | No |  |

*From:*  Moher D, Liberati A, Tetzlaff J, Altman DG, The PRISMA Group (2009). Preferred Reporting Items for Systematic Reviews and Meta-Analyses: The PRISMA Statement. PLoS Med 6(7): e1000097. doi:10.1371/journal.pmed1000097

For more information, visit: **www.prisma-statement.org**.

Page 2 of 2
