# Supplementary material for: The Effect of Dexmedetomidine on Emergence Agitation or Delirium in Children After Anesthesia—A Systematic Review and Meta-Analysis of Clinical Studies
Source: Front Pediatr. 2020 Jul 14;8:329. doi: 10.3389/fped.2020.00329 (PMC7381209; doi:10.3389/fped.2020.00329)
Supplement: Supplementary Table 2 — The Newcastle-Otawa Quality Assessment Scale (NOS). [file Table_2.DOC]

The Newcastle-Otawa Quality Assessment Scale (NOS)

| **Selection** |  |
| --- | --- |
| 1) Is the case definition adequate? (1 point) | a) Yes, with independent validation ★  b) Record linkage (e.g. ICD codes in database) or self-report with no reference to primary record  c) No description |
| 2) Representativeness of the cases (1 point) | a) Consecutive or obviously representative series of cases ★  b) Potential for selection biases or not stated |
| 3) Selection of Controls (1 point) | a) Community controls ★  b) Hospital controls  c) No description |
| 4) Definition of Controls (1 point) | a) No history of disease (endpoint) ★  b) No description of source |
| **Comparability** |  |
| Comparability of cases and controls on the basis of the design or analysis (2 points) | a) Study controls for_____(Select the most important factor) ★  b) Study controls for any additional factor (This criteria could be modified to indicate specific control for a second important factor.) ★ |
| **Exposure** |  |
| 1) Ascertainment of exposure (1 point) | a) Secure record (eg surgical record) ★  b) Structured interview where blind to case/control status ★  c) Interview not blind to case/control status  d) Written self report or medical report only  e) No description |
| 2) Same method of ascertainment for cases and controls (1 point) | a) Yes ★  b) No |
| 3) Non-response rate (1 point) | a) Same rate for both groups ★  b) Non respondents described  c) Rate different and no designation |
