# Supplementary material for: The Effect of Dexmedetomidine on Emergence Agitation or Delirium in Children After Anesthesia—A Systematic Review and Meta-Analysis of Clinical Studies
Source: Front Pediatr. 2020 Jul 14;8:329. doi: 10.3389/fped.2020.00329 (PMC7381209; doi:10.3389/fped.2020.00329)
Supplement: Supplementary Table 3 — The bias risk of CCTs by Newcastle-Otawa Quality Assessment Scale (NOS). [file Table_3.DOC]

| **Study** | **Number of stars** | **Result of quality assessment** |
| --- | --- | --- |
| Mason 2011 | 8 | High |
| Jiang 2015 | 5 | Low |
| Keles 2017 | 7 | High |
| Riveros 2017 | 8 | High |
| Long 2018 | 5 | Low |
