# Supplementary material for: The Effect of Dexmedetomidine on Emergence Agitation or Delirium in Children After Anesthesia—A Systematic Review and Meta-Analysis of Clinical Studies
Source: Front Pediatr. 2020 Jul 14;8:329. doi: 10.3389/fped.2020.00329 (PMC7381209; doi:10.3389/fped.2020.00329)
Supplement: Supplementary Table 4 — Meta-regression (dexmedetomidine vs. placebo): estimate of between-study variance % residual variation due to heterogeneity. [file Table_4.DOC]

Meta-regression (dexmedetomidine versus placebo): estimate of between-study variance % residual variation due to heterogeneity.

|  | Coef. | Std. Err. | t | P>|t| | [95% Conf. Interval] | |
| --- | --- | --- | --- | --- | --- | --- |
| Year | -.0077299 | .0360526 | -0.21 | 0.832 | -.0809976 | .0655378 |
| Study methods | .8408136 | .4323474 | 1.94 | 0.060 | -.037822 | 1.719449 |
| Country | .0828187 | .051652 | 1.60 | 0.118 | -.0221507 | .1877882 |
| Time of administration onset | .3821614 | .3622493 | 1.05 | 0.299 | -.3540178 | 1.118341 |
| Type of surgery | -.0551922 | .0660419 | -0.84 | 0.409 | -.1894055 | .0790211 |
| Routes of drug administration | .1513797 | .1362509 | 1.11 | 0.274 | -.1255156 | .4282749 |
| Bias risk of study | .2594755 | .1565548 | 1.66 | 0.105 | -.0571865 | .5761376 |
| Range of age | .1350992 | .0702534 | 1.92 | 0.062 | -.0070018 | .2772002 |
