# Supplementary material for: The Effect of Dexmedetomidine on Emergence Agitation or Delirium in Children After Anesthesia—A Systematic Review and Meta-Analysis of Clinical Studies
Source: Front Pediatr. 2020 Jul 14;8:329. doi: 10.3389/fped.2020.00329 (PMC7381209; doi:10.3389/fped.2020.00329)
Supplement: Supplementary Table 5 — Meta-regression (dexmedetomidine vs. midazolam): estimate of between-study variance % residual variation due to heterogeneity. [file Table_5.DOC]

Meta-regression (dexmedetomidine versus midazolam): estimate of between-study variance % residual variation due to heterogeneity.

|  | Coef. | Std. Err. | t | P>|t| | [95% Conf. Interval] | |
| --- | --- | --- | --- | --- | --- | --- |
| Year | -.0581595 | .0921868 | -0.63 | 0.556 | -.2951333 | .1788143 |
| Study methods | .3615836 | 1.090749 | 0.33 | 0.754 | -2.442277 | 3.165444 |
| Country | -.0786418 | .2642928 | -0.30 | 0.778 | -.7580282 | .6007446 |
| Time of administration onset | .0859804 | .5159119 | 0.17 | 0.874 | -1.240213 | 1.412174 |
| Type of surgery | .0217639 | .0946252 | 0.23 | 0.827 | -.2214779 | .2650057 |
| Routes of drug administration | -.0296369 | .2770657 | -0.11 | 0.919 | -.741857 | .6825833 |
| Bias risk of study | .4648984 | .3858939 | 1.20 | 0.282 | -.5270735 | 1.45687 |
| Range of age | .1677991 | .2849204 | 0.59 | 0.582 | -.5646122 | .9002103 |
